# Human Papillomavirus among Women Undergoing Papanicolaou Smear Test in the Department of Gynaecologic Oncology of a Tertiary Care Centre

**DOI:** 10.31729/jnma.8363

**Published:** 2023-12-31

**Authors:** Mahendra Raj Shrestha, Ajaya Basnet, Rajendra Maharjan, Sagar Ghimire, Nisha Khatri, Arju Shrestha, Lochan Karki, Saujanya Karmacharya

**Affiliations:** 1 Department of Clinical Laboratory, Nepal Armed Police Force Hospital, Balambu, Kathmandu, Nepal; 2 Department of Microbiology, Nepal Armed Police Force Hospital, Balambu, Kathmandu, Nepal; 3 Department of Gynaecologic Oncology, Nepal Armed Police Force Hospital, Balambu, Kathmandu, Nepal; 4 Department of Medicine, National Academy of Medical Sciences, Mahaboudha, Kathmandu, Nepal

**Keywords:** *cytology*, *histology*, *human papillomavirus*, *Nepal*, *prevalence*

## Abstract

**Introduction:**

In invasive cervical specimens or precursors, high-risk human papillomavirus Deoxyribonucleic acid may be detected to identify females at risk of developing cervical cancer. This study aimed to find out the prevalence of human papillomavirus among women undergoing Papanicolaou smear tests in a tertiary care centre.

**Methods:**

A descriptive cross-sectional study was conducted among women undergoing the Papanicolaou smear test in the Department of Gynaecologic Oncology, Nepal Armed Police Force Hospital, between 1 June 2022 and 15 November 2022. Ethical approval was obtained from the Ethical Review Board. A convenience sampling method was used. The point estimate was calculated at a 95% Confidence Interval.

**Results:**

Among the 199 women, 6 (3.02%) (0.64-5.40, 95% Confidence Interval) had human papillomavirus infection. The mean age of the infected females was 31.17±5.57 years. Human papillomavirus DNA for 16 and 18 were detected in 4 (66.67%) and 2 (33.33%) females, respectively.

**Conclusions:**

The prevalence of human papillomavirus in females was found to be lower than other studies done in similar settings.

## INTRODUCTION

Human papillomaviruses, the double-stranded DNA papovaviruses, cause cervical cancer-the second most common and the fifth most lethal cancer in women worldwide.^1,2^ In 2020, global cervical cancer incidence and mortality rates were estimated at 13 per 100,000 women and 7 per 100,000 women, respectively, compared to 16 and 11 per 100,000 women, in Nepal, making it an extremely dangerous disease.^3^

Although cervical cancer is highly prevalent in Nepal, very few studies have evaluated the prevalence of HPV in precursor lesions and invasive cancer. As Nepal has subtle regional and cultural differences, it is vital to describe the prevalence of HPV in cervical intraepithelial lesions and invasive cancer in multiple representative populations to generalize these statistics in cancer prevention programs across the country. However, scarce literature exists concerning cervical cancer in Nepal.

This study aimed to find out the prevalence of human papillomavirus among women undergoing Papanicolaou smear tests in a tertiary care centre.

## METHODS

A descriptive cross-sectional study was conducted among women undergoing the Papanicolaou smear test in the Department of Gynaecologic Oncology, Nepal Armed Police Force Hospital, between 16 May 2022 and 15 November 2022. Ethical approval was obtained from the Ethical Review Board (Reference number: 20790202). Individuals who were >18 years old who visited the hospital's Department of Gynaecologic Oncology for routine examinations and giving consent were included in the study. Individuals with incomplete study details and who did not give informed consent were excluded. A convenience sampling method was used. The sample size was estimated using the following formula:


$$\begin{array}{ll} \text{n} & ={\text{Z}}^{2}\times \frac{\text{p}\times \text{q}}{{\text{e}}^{\text{2}}}\\ & =1.96^{2}\times \frac{0.50\times 0.50}{0.07^{2}}\\ & =196 \end{array}$$

Where,

n = required sample size
Z = 1.96 at 95% confidence interval (CI)
p = prevalence taken as 50% for maximum sample size calculation
q = 1-p
e = margin of error, 7%

The calculated minimum required sample size was 196. However, a total of 199 patients were included in the study.

The patient information sheet recorded demographic, cytological and molecular (HPV-16, HPV-18, and others) findings. The informed consent of participants was obtained, and records that were missing or ambiguous were clarified by contacting involved healthcare workers and/ or the participants themselves. The gynecologic oncology team collected specimens from the cervical os using an endocervical swab, fixed them with a fixative spray, and sent them to the cytohistology laboratory for cytohistological analysis. A fixed smear was prepared in the cytohistology laboratory and immersed in tap water for 3 minutes. Excess water was blotted off the slide. For 60 seconds, the slide was dipped in rapid-pap nuclear stain (hematoxylin solution). Next, the slide was washed after ten seconds by adding three drops of Scottie's tap water buffer. Following that, the slide was immersed for 30 seconds in rapid-pap dehydrant (propranolol) with two solution changes. The slide was dipped in a working cytoplasm stain (og-6 solution, light green SF-eosin) for 45 seconds. The slide was washed in tap water, and excess water was blotted away. Afterwards, a rapid-pap dehydrator was used to dehydrate the slide for 30 seconds and air-dry. The slide was then dipped in xylene, dried, and mounted with dibutyl phthalate polystyrene xylene using a coverslip. Lastly, the smear was reported according to the revised 2001 Bethesda system.^4^ HPV DNA testing was performed only on smears with abnormal epithelial cells. Gynecologic oncologists collected cells from the uterine cervix in a sample collection buffer (5 ml) and sent them to the molecular laboratory for HPV DNA testing. DNA extraction kits were used as directed by the manufacturer (SpinStarTM Viral Nucleic Acid Kit 1.0, ADT Biotech). The Human Papillomavirus Nucleic Acid Amplification Test Kit (Fluorescent Probe-Based RealTime PCR Assay) (Solis BioDyne) was used to detect HPV-16, HPV-18, and other types. HPV positivity was assessed by real-time polymerase chain reaction.

Data were collected and entered in Microsoft Excel 2010 and analyzed in IBM SPSS Statistics version 17.0. Point estimate and 95% CI were calculated.

## RESULTS

**Figure 1:**
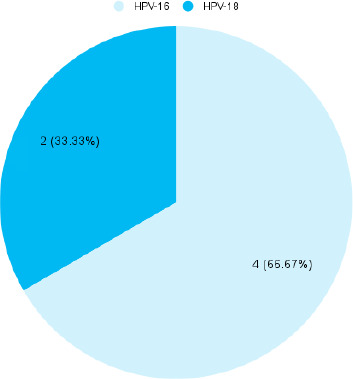
Human papillomavirus genotypes (n= 6).

Among 199 women, HPV infections were detected in 6 (3.02%) (0.64-5.40, 95% CI). HPV-infected women had a mean age of 31.17±5.57 years. Among the females infected with HPV, DNA for genotype 16 was detected in 4 (66.67%) (Figure 1).

A total of 2 (33.33%) cases of low-grade squamous intraepithelial lesions (LSIL) and 2 (33.33%) cases of high-grade squamous intraepithelial lesions (HSIL) were observed among females infected with HPV-16. On the other hand, 1 (16.67%) case of HSIL and 1 (16.67%) case of squamous cell carcinoma (SCC) were observed among females infected with HPV-18.

## DISCUSSION

The prevalence of HPV infections in females in this study was 6 (3.02%), which was lower than in studies conducted in India (15-85%).^5^ This study involved hospital-visiting females, often paramilitary, rather than community-residing females, as in other studies, which resulted in a lower prevalence of HPV infections. A previously published study from Nepal showed higher HPV-16 (72.20%) infection rates than HPV-18 (14.80%), though with varying incidence rates.^6^ A variety of factors influence HPV prevalence, including age of sexual debut, number of partners, parity, use of oral contraception, and socioeconomic status.^7^

In our study, the mean age of the infected females was 31.17±5.57 years. Furthermore, a study involving 361 cervical specimens found that high-risk HPV was most prevalent in females between the ages of 15 and 35 years.^8^ Researchers have linked HPV infection in females to an early age of intercourse, an insufficient adaptive immune response, and relative incompetence in viral clearance because of hormonal changes during the menopausal transition, which may lead to latent HPV infection persistence or reactivation.^9^

The present study showed the highest number of HSIL cases among those infected with HPV, followed by LSIL (33.33%) and SCC (16.67%). Similarly, studies conducted in low- and middle-income countries have shown higher cases of LSIL (41-77.7%) than HSILs (5.2-14%).^10,11^ In this study, it was equally predominant to detect HPV-16 in females with LSIL and HSIL, and HPV-18 in females with HSIL and SCC. Most likely, this is due to cultural differences, including age at marriage, number of sexual partners, religious practices such as circumcision, and personal hygiene.^12,13^ Studies have also shown that females over 40 years have the highest incidence of invasive cancer, which peaks around age 50 and precursors are detectable for up to 10 years before cancer develops, with a peak lesion rate at 35 years of age.^12^

As this study involved only one centre, no generalization of the study findings could be made for the entire nation, therefore further studies involving multiple centres and a larger number of cervical specimens are required.

## CONCLUSIONS

The prevalence of HPV infections in females was lower than in national and international studies performed in similar settings. Formulation of cervical cancer prevention guidelines including screening, and HPV vaccinations could be promoted to decrease the burden of cervical cancer.
